# Building on the Health Policy Analysis Triangle: Elucidation of the Elements

**DOI:** 10.12669/pjms.39.6.7056

**Published:** 2023

**Authors:** Aysha Zahidie, Saima Asif, Meesha Iqbal

**Affiliations:** 1 Aysha Zahidie, MBBS, FCPS (Pakistan) Aga Khan University, Karachi - Pakistan; 2 Saima Asif, MBBS, MCPS (Community Medicine) Army Medical College, Rawalpindi, Pakistan; 3 Meesha Iqbal, MBBS MD MPH FCPS (Community Medicine) UTHealth School of Public Health Houston, Houston, USA

**Keywords:** Health Policies, Policy Analysis, Policy Development, Health Policy Triangle

## Abstract

The health policy triangle first presented in the 20th century by Walt and Gilson has been extensively used at local, national, regional, and international levels to assess health policies related to communicable and non-communicable diseases, physical and mental health, antenatal and postnatal care, and human resources, services, and systems. However, the framework lacks intricate details for the four pillars in the triangle viz: ‘content,’ ‘context’, ‘actors’, and ‘processes. We propose a checklist of elements to be considered for each pillar; to ease and enhance the process of policy analyses for researchers and policymakers across the globe, including low- and middle-income countries. We suggest using Leichter’s categorization of situational, structural, cultural, and environmental factors for comprehensive contextual assessment. Kingdon’s multiple streams framework can be applied to determine the ‘window of opportunity’ allowing the politics, policy, and problem streams to unite, giving birth to the formulation of policies. Lastly, stakeholders’ analyses expounding the power, influence, interest, and involvement of intrinsic, extrinsic, implicit, and explicit players should be applied to explore the ‘actors’ in policy analyses. Robust policy analyses for generating evidence are of paramount importance for policymakers for informed decision-making. Our approach of dis-entangling and elaborating the pillars of the triangle will be helpful for health systems researchers at sub-national, national, regional and global levels to serve as a basis for evidence-based informed decision-making.

**HIGHLIGHTS**

Our approach of dis-entangling and elaborating pillars of the health policy triangle is helpful for health systems researchers to generate relevant information as the basis of evidence-based and informed decision-making.

- Leichter’s categorization is helpful for comprehensive contextual assessment of situational, structural, cultural, and environmental factors affecting policy formulation
- Kingdon’s multiple streams framework can be applied to determine the ‘window of opportunity’ allowing the politics, policy, and problem streams to unite, giving birth to the formulation of policies.
- Stakeholders’ analyses expounding the power, influence, interest, and involvement of intrinsic, extrinsic, implicit, and explicit players should be applied to explore the ‘actors’ in policy analyses.

Policy is a purposive set of actions followed by an actor (usually the government) in dealing with a problem or a matter of concern.^1^ A health policy is an agreement or consensus on the health issues, goals and objectives to be addressed, the priorities among those objectives, and the main directions for achieving them.^2^ Policy expression can take theoretical and empirical forms. Regulations and legislation; guidelines, standards, and targets form formal manifestations of policies, which set out national and/or sub-national priorities, basis for international agreements, and provide a framework for resource allocation and a benchmark for gauging accountability. Policies can be exhibited more subtly through general statements about organizational or national priorities, the funded programs of a country, and unwritten but widely accepted practices and traditions. Policy analysis refers to a wide range of techniques used to holistically scrutinize the features of established policies, their evolution, and consequences considering given goals and targets.^3^

Policy analysis is a multi-disciplinary inquiry that explains reasons for the success or failure of policies and provides a roadmap for further planning. It helps the decision-makers to plan by making informed choices about the context and involvement of implicit and explicit actors in the process of policy instigation. The chaotic response to Covid-19 has underscored the inadequate preparedness of health systems and emphasized that countries should systematically evaluate and disseminate evidence about what works and what does not, to implement new, and scale up previously proven innovations to improve people’s health and survival probability.^4^ Policy analysis is a pragmatic tool to establish clear linkages between health systems’ functions and outcomes, improve the quality of decisions, and guide the development of evidence-based, realistic, and achievable health plans and strategies, including responding and recovering from the pandemic and preparing systems for future crisis. Conducting a policy analysis attests that the policymakers have gone through a scientific process to strategize future steps. Effective policy analysis, whether to implement new policies or revise existing ones, is a critical decision-making tool in the policy process.

Numerous frameworks and approaches to policy analyses have been coined in the past several decades.^5^-^9^ We elaborate the technique by building on the health policy triangle (HPT), first presented by Walt and Gilson in 1994 and used immensely thereafter.^10^ According to a recent systematic review, the HPT has been ubiquitous in the health policy literature to analyze a large number of health-related policy concerns, especially in low and middle-income countries.^11^ Its universal nature allows it to be applied to national, regional, and global levels. However, many researchers complement the HPT with additional frameworks and tools to refine and elaborate their analyses. There is a consensus that there is a dearth of conceptual and theoretical approaches and frameworks to comprehensively assess the processes of HPT.^12^ We intend to fill this literature gap by elaborating on the elements that need to be assessed for each pillar of HPT. Fig.1 demonstrates HPT,^13^ according to which the policy analyses hangs on four crucial pillars of assessing the (a) content, (b) context in which the policies originate, (c) the processes, and (d) actors negatively or positively involved in the formulation and implementation of policies. We shall discuss each component in detail.

**Fig.1:**
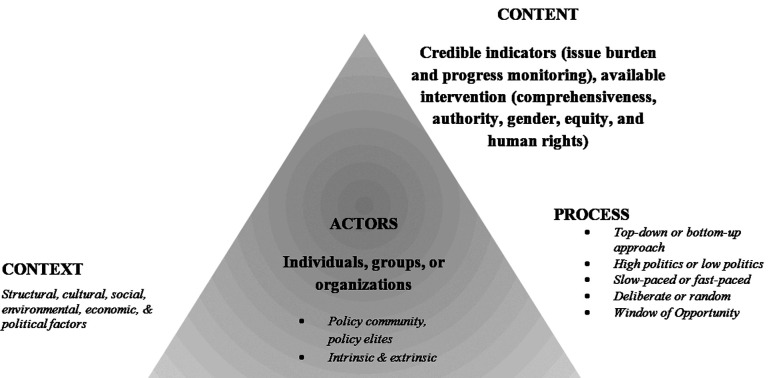
The Policy Analysis Triangle, adapted from Walt and Gilson 1994.^8^

“Context” refers to the complex ecosystem that influences policy dialogue and decisions. Context matters as it influences the degree to which the policy community agrees on the definition, causes, and solutions to the problem as well as public portrayals of the issue in ways that resonate with external actors, especially the political leaders who control resources.^14^,^15^ Leichter’s categorization of contextual factors assesses the situational, structural, cultural, and environmental factors that affect the policy processes (Box-1).^16^ Nonetheless, the list is not exhaustive and may include other relevant factors such as economic and political elements, depending upon the problem under consideration. These factors build up political moments when conditions align favorably for an issue, presenting opportunities for advocates to influence decision-makers. Moreover, they reflect the degree to which norms and institutions operating in a sector provide a platform for effective collective action.

“Process” refers to the way a policy is instigated; it could follow a top-down or bottom-up approach, be slow-paced or fast-paced, deliberate or random, smooth or messy. Policy processes could be ‘high politics’ requiring fundamental changes in the role of the state (e.g., travel restrictions amidst Covid-19), or ‘low politics’ that do not question the status quo.^10^ Kingdon’s Multiple Streams Framework delineates that the policy process is embedded in the problem, policy, and politics stream (Fig.2). The three streams conventionally run independently; the window of opportunity opens when all three come together and the policy emerges.^17^ Many other models for assessing policy processes exist: the rational approach^18^ considers policy making a linear process and is rarely witnessed in the real world; the ‘incrementalism’ approach^19^ reflects the process as bargaining, negotiation, and adjustment between the stakeholders, ‘mixed scanning’^20^ includes taking a bird’s eye view of the policy arena and randomly picking up operational areas, ‘diffusion *of* innovations theory’^21^ relates to transfer of international agendas into national agendas, and ‘path dependency model’^22^ exhibits replication of past policy agendas into future directions.^23^ The policy process often follows a muddling through non-linear, irrational route, influenced by myriad push and pull factors. Policy analysts need to consider all aspects of the policy process to develop a comprehensive understanding of the course of policy emergence.

**Fig.2:**
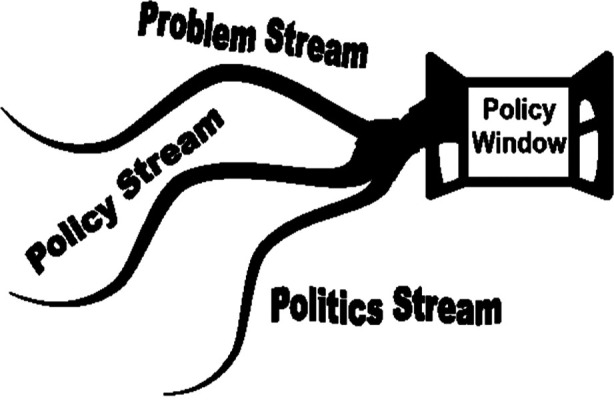
The Kingdon’s Three Streams Policy Window Model.^17^

The actors could be individuals, groups, or organizations involved in the policy reform process. These include intrinsic and extrinsic, explicit, and implicit players; sub-national, national, or international agencies, multi-lateral, bi-lateral, and non-governmental organizations forming policy elites, communities, and issue networks. Simultaneously, the power and influence of street-level bureaucracy should not be overlooked in policy analyses. Policy analysts should thoroughly analyze and appreciate the position, power, interests, and characteristics of the involved stakeholders and alliances to understand the policy landscape (stakeholder analyses).

The strength of actors and organizations concerned with the issue could form an important and interesting arena for policy analysis to identify if those who are going to be affected by the policy have any role to play in policy formulation. When issues are not controversial, the framing of actors could still be an issue (Shiffman & Smith framework).^24^Actors also need to be analyzed in the context of presence or absence of guiding institutions with any mandate to lead the initiative. Additionally, the degree of agreement and cohesion or disagreement and distrust among various actors is also important to be analyzed while assessing the policy formulation process.

Policy content analysis is the process of describing policies quantitatively (in terms of numbers) and qualitatively. Quantitative content analysis should ideally be based on issue characteristics explained through credible indicators expressing the severity of the problem on one hand and progress monitoring on the other, as well as reflecting the problem burden relative to other existing conditions. It should also entail effective interventions meant to address the problem, in the most cost-effective and scientifically proven manner. Policy content review may include a policy cube approach for qualitative analysis in HPT, discussing the comprehensiveness of global best buys on included interventions, and other axes including human rights, gender, and equity along with the depth of authority for implementation.^25^

## Future Research and Practical Implications:

Policy analysis is an arduous task. Explicit blueprints may not be available to determine the terrain of policies. It is important to scrupulously analyze all the processes, contextual factors, and involved actors along with the content of policies for a comprehensive understanding of policy reforms. The elements of each pillar (content, context, processes, actors) as detailed in Fig.1 and Box-1 need to be kept in mind before commencing policy analyses. Nonetheless, the list is not exhaustive and amenable to change according to the context and nature of the health concern. The stakeholders ought to utilize policy analyses as a decision-making tool to avoid past errors, make a cautious selection and adopt the best measures for future implementation.

## Author Contribution:

**MI:** Conception and design of the work. Is responsible and accountable for the accuracy and integrity of the work.

**MI, SA,** and **AZ:** Data collection/Literature search.

**NA:** Data analysis and interpretation.

**MI, SA,** and **AZ:** Drafting the article.

**MI, S A,** and **AZ:** Critical revision of the article.

**MI, SA,** and **AZ:** Final approval of the version to be submitted- all named authors should approve the paper before submission.

Box 1: Leichter’s categorization of contextual factors in policy analysis.^16^

***Situational factors***- idiosyncratic, transient conditions, e.g., disasters, Covid-19, change of government etc.

***Structural factors:*** permanent characteristics of health systems or policy apparatus, e.g., devolution, health-care delivery system etc.

***Cultural factors:*** societal, organizational, and individual beliefs and values e.g., resistance to mask wearing in the United States amidst Covid-19.

***Environmental factors:*** external/ international values, events, agreements, and structures e.g., across border travel restrictions during Covid-19.

***Environmental factors***: external/ international values, events, agreements, and structures e.g., across border travel restrictions during Covid-19.

**Box 2:** Use of Health Policy Triangle in Policy Analysis

**“Analysis of alcohol policy in Nigeria: multi-sectoral action and the integration of the WHO “best-buy” interventions”^26^**

This study demonstrated the use of HPT to investigate alcohol related policies in Nigeria against the backdrop of reducing risk factors of non-communicable diseases. One of the recommendations given by authors was to include the “multiple streams” framework, for robust analysis. Our proposed framework enhances the HPT by incorporating the multiple streams framework.

**“Policies and Programs for Prevention and Control of Diabetes in Iran: A Document Analysis”^27^**

This study examined the policies and programs related to diabetes control in Iran using HPT. The authors disentangled the HPT and elaborated the “process” by detailing *agenda setting, formulation, implementation and evaluation* of diabetes related programs. We propose the inclusion of *multiple streams framework* to understand challenges and opportunities related to diabetes control more comprehensively. Similarly, the pillars of “context” and “actors” could have been more scientifically elaborated by utilizing the Leichter’s framework and intrinsic and extrinsic stakeholders.

***“Retaining Doctors in Rural Bangladesh: A Policy Analysis”^28^***

This study assessed the policies to retain doctors in rural Bangladesh using HPT. The authors have utilized “stakeholders’ analysis” to analyze *actors* and discussed the implementation issues under *process* of HPT.
